# Hospital-based or home-based administration of oncology drugs? A micro-costing study comparing healthcare and societal costs of hospital-based and home-based subcutaneous administration of trastuzumab

**DOI:** 10.1016/j.breast.2020.05.001

**Published:** 2020-05-16

**Authors:** Margreet Franken, Tim Kanters, Jules Coenen, Paul de Jong, Agnes Jager, Carin Uyl-de Groot

**Affiliations:** a Institute for Medical Technology Assessment, Erasmus University Rotterdam, the Netherlands; b Erasmus School of Health Policy & Management, Erasmus University Rotterdam, the Netherlands; c Department Oncology, Isala, Zwolle, the Netherlands; d Department of Internal Medicine, St. Antonius Hospital, Utrecht, the Netherlands; e Department of Medical Oncology, Erasmus MC Cancer Institute, Rotterdam, the Netherlands

**Keywords:** Breast cancer, Healthcare costs, Home-based, Hospital-based, Injection, Micro-costing, Netherlands, Resource use, Societal costs, Subcutaneous, Trastuzumab

## Abstract

**Objective:**

To investigate resource use and time investments of healthcare professionals, patients and their family and to compare healthcare and societal costs of one single hospital-based and one single home-based subcutaneous administration of trastuzumab in The Netherlands.

**Method:**

We conducted a bottom-up micro-costing study. Patients diagnosed with HER2+ early or metastatic breast cancer were recruited in four Dutch hospitals. For healthcare costs, data were collected on drug use, consumables, use of healthcare facilities, time of healthcare professionals, and travelling distance of the nurse. For societal costs, data were collected on patient and family costs (including travelling expenses and time of informal caregivers) and productivity losses of paid and unpaid work.

**Results:**

Societal costs of one single administration of SC trastuzumab were €1753 within the home-based and €1724 within the hospital-based setting. Drug costs of trastuzumab were identical in both settings (€1651). Healthcare costs were higher for home-based administration (€91 versus €47) mainly because of more time of healthcare professionals (110 versus 38 minutes). Costs for patient and family were, however, lower for home-based administration due to travelling expenses (€7 versus €0) and time of informal caregivers (€14 versus €4). Costs for productivity losses were similar for both settings.

**Conclusions:**

Home-based subcutaneous administration of trastuzumab is more time consuming for healthcare professionals and therefore more costly than hospital-based administration. The total budget impact can be large considering that a large number of patients receive a large number of cycles of oncology treatments. If home-based administration is the way forward, novel approaches are crucial for ensuring efficiency of home-based care.

## Highlights

• Home-based administration of SC trastuzumab almost triples the time of healthcare professionals.
• Healthcare costs are almost twice as much for home-based than for hospital-based administration.
• Patient and family costs are almost five times lower for home-based than for hospital-based administration.

## 1 Introduction

Worldwide, about 2.1 million women are yearly diagnosed with breast cancer, which is a quarter of all new cancer cases in women [1]. In 2013, the European Medicines Agency (EMA) approved the subcutaneous (SC) formulation of trastuzumab (Herceptin®) for patients with HER2+ breast cancer based on similar pharmacokinetic, efficacy and safety compared to intravenous (IV) trastuzumab [2].

Several studies showed high patient satisfaction and preferences for SC over IV administration within the hospital setting mainly because of time savings for patients and healthcare professionals [[3], [4], [5]]. These time savings resulted in lower healthcare as well as lower societal costs for SC compared to IV administration [[6], [7], [8], [9]]. Patient satisfaction and preferences may even be higher in case the SC injections will be administered at home. Especially in the context of the growing pressure on the capacity of oncology day-care units and healthcare policy of shifting hospital to homecare, it can be expected that an increasing number of patients will receive home-based care. A recent systematic review [10] showed, however, that there is only limited evidence on time investments of patients and healthcare professionals and the associated costs of home-based administration of oncology drugs.

The aim of this study was to investigate resource use and time investments of healthcare professionals, patients and their family and the associated costs of the SC administration of trastuzumab in The Netherlands. We compared healthcare and societal costs of one single hospital-based and one single home-based SC administration of trastuzumab.

## 2 Methods

### 2.1 Study design

We conducted an observational non-interventional study using a bottom-up micro-costing approach, which is the ‘gold standard’ for costing studies [11]. The following three cost categories were included: i) costs within the healthcare sector including drugs, use of healthcare facilities, time of healthcare professionals, consumables, and, for home-based administration, travelling expenses of healthcare professionals; ii) patient and family costs including travelling expenses of the patients and time of family and/or friends; and iii) costs in other sectors, in particular costs related to productivity losses.

### 2.2 Patient population

Patients were recruited in four hospitals (one academic and three general hospitals). All four hospitals administrate SC trastuzumab at the oncology day-care unit. Two of the four hospitals provided home-based administration, both on a pilot project basis. To ensure safe use of trastuzumab, patients only receive home-based administration by a specialised nurse and after at least one administration at the day-care unit. One hospital offered this service to all patients if they had a preference of home-based over hospital-based administration and the other hospital offered this service only to a limited number of patients (at discretion of the oncologist in consultation with the patient). One hospital organised home-based administration by oncology nurses from their day-care oncology unit, the other outsourced this to two homecare organisations for specialised care. Generally, most patients receive one SC administration at the hospital (often on the same day they visit the oncologist) followed by a block of three administrations at home, which is at the discretion of their oncologist in collaboration with the patient. The online Supplementary file shows the care pathways of both settings.

This study is a follow-up of a study [7] in which we compared healthcare and societal costs of IV and SC administration within the hospital setting. Patients were eligible for inclusion if they were 18 years or older and diagnosed with HER2+ early or metastatic breast cancer. For this study, we included all patients receiving hospital-based administration of SC trastuzumab monotherapy (i.e., excluding patients of our previous study who received rituximab, trastuzumab IV, and combination therapy). All patients receiving SC trastuzumab monotherapy were approached for participation when they visited the day-care unit at the days of data collection in the four hospitals. Full details and results of the previous study are reported elsewhere (Franken et al. [7]). For the home-based setting, we recruited new patients. All patients receiving SC trastuzumab monotherapy organised via the two hospitals were approached for participation. As we only included patients who received monotherapy, we did not have to make a distinction between early and metastatic breast cancer as SC trastuzumab monotherapy is used identical in both settings. Informed consent was signed by all included patients. Ethical approval by the medical ethical committee was obtained in the participating hospitals.

### 2.3 Data collection

Data were collected at the hospital pharmacy (preparation of trastuzumab injection), oncology day-care unit (hospital-based administration) and at patients’ home (home-based administration).

Data for healthcare-related costs were collected using case report forms and questionnaires. Regarding the preparation of the injection, data were collected at the hospital pharmacy for 20 SC trastuzumab injections including usage of consumables and time of healthcare professionals needed for preparation and delivery of trastuzumab. Regarding the administration of SC trastuzumab, a case report form was completed for each patient. This included data on usage of consumables, time investments of healthcare professionals (including ‘non-bedside’ activities such as time for reporting and administrative tasks), and for home-based administration travelling distance and travelling time of the nurse (i.e., distance and time from hospital or previous patient to the home of the patient plus the time from the last patient to the hospital divided by the number of patients visited). Besides this, each hospital and homecare organisation completed one questionnaire regarding time investments for related administrative tasks not included in the case report forms (e.g., time for referral and reporting between hospital and homecare organisation, time for scheduling and phone calls to patients).

Data for patient and family costs and productivity losses were collected using patient questionnaires. These questionnaires included questions on age, gender, travel distance to hospital, mode of transportation, paid and unpaid work, sick-leave, and informal care (friend or family member accompanying the patient). The questionnaire for home-based administration also included one question on the expected time of arrival of the nurse (i.e., potential waiting time) and questions on preference and satisfaction regarding administration within both settings (i.e., What do you prefer: hospital- or home-based administration? Why do you prefer this? Can you give a score between 1 and 10 for your level of satisfaction for the hospital setting and home-based setting?).

All data were collected between March 25, 2017 and August 10, 2017 for hospital-based and between January 8, 2018 and July 17, 2018 for home-based administration.

### 2.4 Unit costs and cost analysis

Costs were computed using the methodology as described in the Dutch costing manual [12,13]. Resource use volumes were multiplied with unit prices. Time of healthcare professionals was valued using wage rates. Unit costs for consumables were provided by the participating day-care units and, if unavailable, complemented with market prices. Costs for the preparation of trastuzumab consisted of costs for consumables and the required time of healthcare professionals measured at the hospital pharmacy. In cases where trastuzumab was administered by nurses of the homecare organisations, the injection preparation time was included in the total nurse time at patients’ home (injections are prepared by nurses of the homecare organisation at a patient’s home). Costs of trastuzumab were obtained from the Z-index (i.e., official Dutch list-price without VAT), version October 2017.

Reference prices of the Dutch costing manual [12,13] were used for valuing productivity losses, travelling expenses and informal care. Productivity losses were valued according to the friction-cost methodology (i.e., absence from work because of illness and/or treatment restricted to a friction period of 12 weeks; after this friction period no further productivity costs are incurred as it is assumed that another worker has fully replaced the person who is absent due to illness [14]).

Overhead costs for hospital-based administration were determined using the financial administration of oncology day-care units (including costs for infrastructure and other facility-associated costs). The actual time spent on the day-care unit was multiplied with the unit cost per minute of admission (i.e., €0.76 per minute; see Franken et al. [7] for further details). As patients do not spent time in the day-care unit in case of home-based administration and because no data was available for overhead costs of the homecare organisations, overhead costs related to home-based administration were determined using information from annual financial statistics of Dutch Hospital Data [15]. Although these aggregated data are not specific for the participating hospitals, it distinguishes different cost categories allowing separating direct costs and indirect costs relevant to the homecare setting. As such, we could determine a mark-up percentage on direct costs (i.e., 19.7%). Overhead costs were then computed by multiplying all direct costs with this mark-up.

All costs were based on Euro 2017 prices; where necessary unit costs were adjusted to 2017 prices using the general price index from the Dutch Central Bureau of Statistics [16]. Table 1 presents the unit costs. Patient baseline characteristics were summarized using descriptive statistics. Statistical significance of the differences between patients receiving home-based and hospital-based administration was assessed using an independent sample *t*-test for age and using Pearson’s chi-square test for the presence of an informal caregiver, paid and unpaid work.

**Table 1:** **Unit costs**.

	Unit costs (Euro 2017 prices)	Source
**Time of healthcare professionals**		
Pharmacist	€ 0.87 per minute	Wage rates
Hospital care: Nurse	€ 0.56 per minute	Wage rates
Homecare: Nurse	€ 0.58 per minute	Wage rates
Pharmacy employee	€ 0.41 per minute	Wage rates
Pharmacy assistant	€ 0.46 per minute	Wage rates
Hospital: planner	€ 0.46 per minute	Wage rates
Homecare organisation: planner	€ 0.39 per minute	Wage rates
**Overhead costs**		
Hospital care: Time spent at day-care unit	€ 0.76 per minute	Financial administration of daycare units
Homecare: Overhead mark-up	19.7%	DHD data
**Drug costs (Source: Z-index, Oct. 2017)**		
Trastuzumab 600 mg (injection)	€ 1651.41	Z-index
**Societal costs**		
Reimbursement for travelling distance nurses		Financial administration of hospital
*Car*	€ 0.30 per km	
Travelling expenses patients		Hakkaart et al.
*Car*	€ 0.19 per km + € 3.07 parking costs	
*Public Transport*	€ 0.19 per km	
*Biking/Walking*	€ 0.00 per km	
Informal care costs	€ 14.32 per hour	Hakkaart et al.
Paid work	€ 32.33 per hour (female)	Hakkaart et al.
Unpaid work	€ 14.32 per hour	Hakkaart et al.
**Consumables (per item)**		Financial administration of daycare units
Subcutaneous needles 25 Gauge	€ 0.11	
Blunt fill needle	€ 0.13	
Syringe 5 mL	€ 0.05	
Sterile (non-sterile) gloves (pair)	€ 0.48 (€ 0.06)	
Sterile (non-sterile) gauze 5 × 5 & 10 × 10 cm	€ 0.015 (€ 0.003) & € 0.015 (€ 0.007)	
Non-sterile protective coating	€ 0.09	
Disinfectant/alcohol wipe	€ 0.12	
Gown & hat	€ 0.11 & € 0.06	

All time measurement, resource use and costs were reported per single administration of SC trastuzumab as averages including standard deviation (SD). As time and cost data are typically skewed and non-normal distributed, classical hypotheses tests (e.g., Student *t*-test) are not appropriate. Therefore, statistical significance of the difference in time investment and costs between home-based and hospital-based administration of SC trastuzumab was assessed by using non-parametric bootstrap techniques as described by Desgagné et al. [17] in Microsoft Excel®, version 2013. Using this method, statistical tests are performed on a large number of bootstrapped samples (i.e., 1000) which are drawn from the observed data. All other statistical analyses were conducted using the statistical software of STATA®, version 14.1 (StataCorp LP. College Station, Texas, USA).

## 3 Results

### 3.1 Patients

In total 68 female patients were included in the study: 39 received hospital-based and 29 received home-based SC trastuzumab. Participation rate was high, only four out of 72 patients could not be included in the study. Of these four patients, two patients did not return the patient questionnaire, one patient could not complete the questionnaire due to a language barrier, and one patient did not consent for participation. Table 2 presents the patient characteristics. About three-quarter of the patients within the home-based setting received the injection by a nurse from the oncology day-care unit. Informal care givers more often accompanied patients in the hospital setting (49% versus 10%; p = 0.001). Although relatively more patients within the hospital-based setting had paid work (67% versus 48%), most patients were absent of work longer than 12 weeks (i.e., no costs for productivity losses as the friction period has been completed).

**Table 2:** **Patient chararcteristics**.

	Home-based administration	Hospital-based administration	p-value ∗
Number of patients	29	39	
Age, mean [SD]	51.6 [14.8]	53.1 [12.0]	0.665
Female	100%	100%	
Homecare organisation	24.1%	n/a	
Accompanying informal caregiver	10.3%	48.7%	0.001
Paid work	48.3%	66.7%	0.128
>12 weeks absent due to illness	85.7%	80.8%	0.695
Unpaid work	16.7%	17.9%	0.896

∗ Statistical significance of the differences was assessed using an independent sample *t*-test for age and using Pearson’s chi-square test for the other characteristics.

### 3.2 Time measurements and travel distances

Table 3 shows the time measurements of healthcare professionals, patients, family and productivity losses and travel distances. Time investments of healthcare professionals was much larger for home-based administration (110 versus 38 min; p = 0.004). This was largely due to travelling time of the nurse (41 min in the home-based setting) and longer time spend with the patient (31 min versus 13 min). Patients stayed on average 62 min at home whereas patients spent 46 min in the hospital (note: excluding travelling time of the patient). The travelling distance was somewhat greater for nurses in the home-based setting (27 km) compared to patients in the hospital-based setting (23 km). There were no productivity losses from paid work, because patients either had no paid work or were absent from work longer than the 12-week friction period. Productivity losses from unpaid work were small and comparable between both groups. Time of informal care givers was almost three times higher within the hospital setting (57 versus 19 min).

**Table 3:** Time measurements for a single SC trastuzumab injection.

*Time in minutes [SD]*	Home-based administration (n = 29)		Hospital-based administration (n = 39)		Difference	P-value^c^
	Mean	SD	Mean	SD		
***Patient***						
Time stayed at home for injection	62.1	53.4	n/a	n/a		
Time spent in hospital (arrival to discharge)	n/a	n/a	45.5	42.2		
Length of stay day-care unit	n/a	n/a	33.1	36.7		

***Hospital***						
**Total Healthcare professional time**	**110.2**	**62.9**	**38.3**	**4.8**	**71.9**	0.004
Preparation of trastuzumab						
Healthcare professional time^a^	n/a^a^	n/a^a^	5.8	3.1		
Administration of trastuzumab						
Responsible nurse	**95.9**	**49.1**	**28.7**	**4.9**	67.2	0.001
Bedside activities (time with patient)	30.7	10.8	12.6	4.9	18.1	
*Time with patient*	26.0	10.9	6.2	4.2	19.8	
*Duration SC injection*	4.7	1.9	6.4	1.9	−1.7	<0.001
Non-bedside activities	23.9	17.3	16.1	12.4	7.7	
Travel time nurse	41.3	32.8	n/a	n/a		
Second nurse	**1.6**	**1.0**	**0.6**	**0.2**	1.0	
Planner in hospital-based setting^b^	**2.7**	**1.3**	**3.2**	**0.9**	−0.5	
Planner in home-based setting	**2.0**	**3.7**	**n/a**	**n/a**		
Referral & reporting (between hospital & homecare organisation)	**8.0**	**14.4**	**n/a**	**n/a**		

***Societal***						
Productivity losses						
Paid work (hours)	0.00	0.00	0.00	0.00	0.00	
Unpaid work (hours)	0.41	1.19	0.38	0.88	0.03	
Time of informal caregiver (hours)	0.31	0.97	0.94	1.13	−0.63	
Distance patient's home to hospital (kilometers)	15.50	13.34	11.54	8.84	3.96	
Travelling distance patient (kilometers)	n/a	n/a	23.08	17.68		
Travelling distance nurse (kilometers)	26.80	28.17	n/a	n/a		

SC subcuta*neous; SD standard deviation*.

a *for hom*e-based administration, preparation time of the trastuzumab injection was included in costs for ready to use trastuzumab injection.

b Costs for the time of a planner were in the hospital-based setting included within the overhead costs.

c Statistical significance of the difference in time was assessed using non-parametric bootstrap techniques (1000 bootstraps).

### 3.3 Healthcare and societal costs

Table 4 presents healthcare and societal costs for a single SC administration of trastuzumab. Drug costs were identical in both settings (€1651). Administration costs for a single injection excluding drug costs were €72.86 for a hospital-based and €101.70 for a home-based administration (difference in costs: €28.84; p = 0.027). Costs for time of healthcare professionals were three times higher within the home-based setting (€63.11 versus €19.52). Overhead costs were, however, lower for home-based administration (€13.71 versus €25.29). Although healthcare costs were higher for home-based administration (€91.33 versus €47.11; p = 0.001), societal costs were higher for hospital-based administration (€25.75 versus €10.37; p = 0.008). This was partly related to a shift from societal costs in the hospital-based setting to healthcare costs in the home-based setting due to travelling expenses (i.e., out-of-pocket expenses for patients shifted to costs for healthcare). The remainder of the difference was due to the greater time informal care givers accompanied patients within the hospital-based setting, resulting in higher (societal) costs of informal care compared to the home-based setting (€13.52 versus €4.44).

**Table 4:** Average healthcare and societal costs for a single SC trastuzumab injection.

*Average costs in Euro (2017)*	Home-based administration (n = 29)	Hospital-based administration (n = 39)	Difference	P-value^c^
**Healthcare costs**				
Active healthcare professional time	€ 63.11	€ 19.52	€ 43.59	
Preparation of trastuzumab^a^	€ -	€ 2.99	€ −2.99	
Administration of trastuzumab^b^	€ 63.11	€ 16.53	€ 46.59	
Consumables	€ 6.48	€ 2.30	€ 4.17	
Preparation of trastuzumab^a^	€ -	€ 1.83	€ −1.83	
Administration of trastuzumab	€ 6.48	€ 0.47	€ 6.01	
Ready to use trastumuzab injection^a^	€ 6.03	€ -	€ 6.03	
Syringes & needles	€ 0.14	€ 0.16	€ −0.01	
Desinfectant, gauzes, bandages & plasters	€ 0.15	€ 0.13	€ 0.02	
Protective materials (e.g. gloves, gown, mask)	€ 0.12	€ 0.18	€ −0.06	
Overhead costs	€ 13.71	€ 25.29	€ −11.58	
Hospital-based administration: time at day-care unit		€ 25.29		
Home-based administration: mark-up on direct costs	€ 13.71			
Reimbursement of travelling expenses (nurse)	€ 8.04	€ -	€ 8.04	
***Subtotal healthcare costs excluding trastuzumab***	**€ 91.33**	**€ 47.11**	**€ 44.22**	**0.001**
*SD*	*€ 46.75*	*€ 29.62*		
*min*	*€ 50.20*	*€ 23.71*		
*max*	*€ 272.36*	*€ 181.75*		
Trastuzumab	€ 1651.41	€ 1651.41	€ -	
***Subtotal healthcare costs including trastuzumab***	**€ 1742.74**	**€ 1698.52**	**€ 44.22**	

**Societal costs**				
Travelling expenses patient	€ -	€ 6.72	€ −6.72	
Time of informal caregiver	€ 4.44	€ 13.52	€ −9.08	
Productivity losses				
Paid work	€ -	€ -	€ -	
Unpaid work	€ 5.93	€ 5.51	€ 0.42	
***Subtotal societal costs***	**€ 10.37**	**€ 25.75**	**€ -15.38**	**0.008**
*SD*	*€ 20.66*	*€ 24.80*		
*min*	*€ -*	*€ -*		
*max*	*€ 71.60*	*€ 98.53*		

**Total costs for a single SC administration**				
Excluding trastuzumab	**€ 101.70**	**€ 72.86**	**€ 28.84**	**0.027**
*SD*	*€ 55.93*	*€ 46.14*		
*MIN*	*€ 50.20*	*€ 29.79*		
*MAX*	*€ 272.36*	*€ 222.96*		
Including trastuzumab	**€ 1753.11**	**€ 1724.27**	**€ 28.84**	
*SD*	*€ 55.93*	*€ 46.14*		
*MIN*	*€ 1701.61*	*€ 1681.20*		
*MAX*	*€ 1923.77*	*€ 1874.37*		

SC sub*cutaenous; SD standard deviation; MIN minimum observed costs; MAX maximum observed cost*.

a *Fo*r home-based administration, costs for the preparation of the injection related to the time of healthcare professionals and usage of consumables were included in costs for ready to use trastuzumab injection.

b Costs of adminstrative staff (planner) in the hospital setting were included in the overhead costs (thus not in the active time of healthcare professionals).

c Statistical significance of the difference in costs was assessed using non-parametric bootstrap techniques (1000 bootstraps).

## 4 Discussion

We investigated resource use and time investments of healthcare professionals, patients, family and productivity losses and the associated costs of the administration of SC injections of trastuzumab. Our study showed that home-based SC administration of trastuzumab costs more than hospital-based administration. Costs for healthcare are higher for home-based administration as it requires more time of healthcare professionals. Costs for patient and family were, however, lower for home-based administration due to travelling expenses and time of informal care givers.

Previous studies showed high patient satisfaction and preferences for SC over IV administration and lower costs for SC administration [[3], [4], [5], [6], [7], [8], [9]]. The shift to home-based SC administrations of trastuzumab is, however, relatively new in The Netherlands [18]. The two hospitals that provided home-based administration, considered this to be of great added value for patients as it is less intensive for patients. Other reasons for providing home-based administration were limited capacity of the oncology day-care unit, collaboration of care across healthcare settings, and requests from patients as well as market authorisation holders. However, home-based administration within these hospitals was provided on a pilot project basis and it should be noted that hospitals do not receive earmarked funding for home-based administration.

To our knowledge, there is limited evidence on healthcare and societal costs of home-based SC administration of oncology drugs. A recent systematic review [10] identified two studies [19,20] comparing costs of hospital-based and home-based SC injections of bortezomib for Multiple Myeloma. In contrast to our study, both studies reported higher costs for hospital-based administration. Both studies, however, calculated costs using flat rates and/or DRG tariffs (e.g., a flat rate for admission day-care unit). In contrast, we conducted a bottom-up micro-costing study and based the costs on the actual measured time of healthcare professionals, usage of consumables, time spent at the day-care unit, and time of informal care givers. Therefore, our cost estimates are more accurate as they are based on actual consumption of healthcare professional time and resources instead of using flat rates (e.g., negotiated prices) identical for every patient irrespective of actual consumption. A bottom-up micro-costing approach can be considered as the ‘gold standard’ for costing studies [11].

It should be noted that our study has some limitations. First, it was impossible to use an identical method for determining overhead costs in both settings. The method used for hospital-based administration is the most preferred method as overhead costs were calculated using financial data of the participating day-care units. This was, however, not possible for home-based administration because i) patients do not occupy a hospital bed for home-based administration, and ii) such data were not available. Therefore, we used a generic mark-up on direct costs for the home-based setting. As overhead costs are accountable for 40% of the difference in costs between hospital-based and home-based administration, we may either have under- or overestimated the difference in costs. For example, if we would have used a mark-up of 30% or 40% instead of 19.7%, the overhead costs for home-based administration would be €20.88 and €27.83 instead of €13.71, respectively. This would increase the difference in costs between home-based and hospital-based administration from €28.84 to €36.02 and €42.97, respectively. Second, some of the differences may be due to the relatively small sample size and the observational nature of our study as patients received care as usual and were not randomly assigned to either hospital-based or home-based administration. Although other observational studies successfully applied statistical techniques to improve comparability of patient groups, essential prerequisites [21], such as large patient numbers and (information on) valid predictors for costing items, were missing in our study.

Even though our study was conducted in The Netherlands and only included a small number of patients, we believe that our study provides important new information which may have implications for healthcare professionals, hospitals, healthcare decision makers and healthcare payers in other countries. As we observed that the differences in costs were mostly related to the additional time of the nurse in the home-based setting (more time with the patient as well as time for travelling), we believe that these findings are transferable to other countries irrespective of the healthcare context. Larger studies and studies in other countries are necessary as cost information on home-based administration is highly relevant because of preferences, high patient satisfaction, and the growing pressure on the capacity of oncology day-care units. Our study confirmed that patients were more satisfied with home-based administration (satisfaction score of 9.2 versus 8.4 on a scale of 10). It should be noted that this score may be influenced by selection bias as this question was only included in the patient questionnaire within the home-based setting (i.e. patients with a preference for home-based administration). Our study also revealed that costs are higher for home-based administrations, mainly because time investments of healthcare professionals almost tripled in our study. Most of the greater time investments of healthcare professionals were related to activities which are non-existing in a hospital setting irrespectively of the context of the country. The actual injection time of SC trastuzumab and subsequent time for observation should not deviate between countries as it is set down in the product label. Active nurse time for observation post injection can be lower in any hospital setting because of possible nurse activities for other patients during the observation period. If in the future patients would be able to safely self-administer SC trastuzumab, for example using a single-use injection device, costs could decrease as long as the costs for such a device would not be larger than the current costs for the time of healthcare professionals. Although travelling distance, time, and unit costs for the time of healthcare professionals is context specific, our detailed breakdown of unit costs and resource use allows easy adaptation of our findings to other settings.

The greater time investments of healthcare professionals and its financial consequences could be a reason that hospitals are reluctant for providing home-based administration. Furthermore, our study illustrated the importance of comparing healthcare and societal costs as we observed a shift from societal to healthcare costs. This is relevant as hospitals and healthcare payers are, in The Netherlands, not financially responsible for societal costs such as travelling expenses of the patients, costs of informal care and productivity losses. Consequently, there is a financial disincentive for home-based administration.

More importantly, administration costs are only a small part of the total costs for oncology care. Regarding the high drug costs, although negotiated drug prices remain confidential, potentially much larger savings can be achieved by switching to, most likely less costly, biosimilar equivalents. The biosimilar equivalents are, however, for the coming years only available for IV infusion (either hospital-based or home-based). Nonetheless, although our previous study [7] showed that IV administration is more expensive than SC administration within the hospital setting (difference for one administration in healthcare costs: €68 and societal costs €22), IV administration within the hospital setting would be less expensive in case drug costs of IV trastuzumab (-biosimilar) would decrease by 8% or more compared to drug costs of SC administration of trastuzumab. This underlines the dilemma between the pressure on the capacity of oncology day-care units, preferences for SC over IV administration, preferences for home-based over hospital-based administration, and the costs of the different strategies. It is, therefore, crucial to focus on novel and efficient approaches of hospital-based and home-based care.

In conclusion, this study shows that home-based SC administration of trastuzumab is more time consuming for healthcare professionals and therefore more costly than hospital-based administration. Although the impact seems rather limited considering the absolute costs for one single administration, the budget impact can be large considering the large number of patients who receive a large number of treatment cycles. Notwithstanding the preferences for home-based care and the reduction of the pressure on the capacity of oncology day-care units, if home-based care is the way forward, novel approaches are vital for ensuring efficiency of home-based care.

## Appendix A Supplementary data

Care pathways in the hospital-based and home-based setting Care pathways in the hospital and home-based setting
