# Supplementary figures and images for: Hospital-based or home-based administration of oncology drugs? A micro-costing study comparing healthcare and societal costs of hospital-based and home-based subcutaneous administration of trastuzumab
Source: Breast. 2020 May 16;52:71–7. doi: 10.1016/j.breast.2020.05.001 (PMC7487951; doi:10.1016/j.breast.2020.05.001)

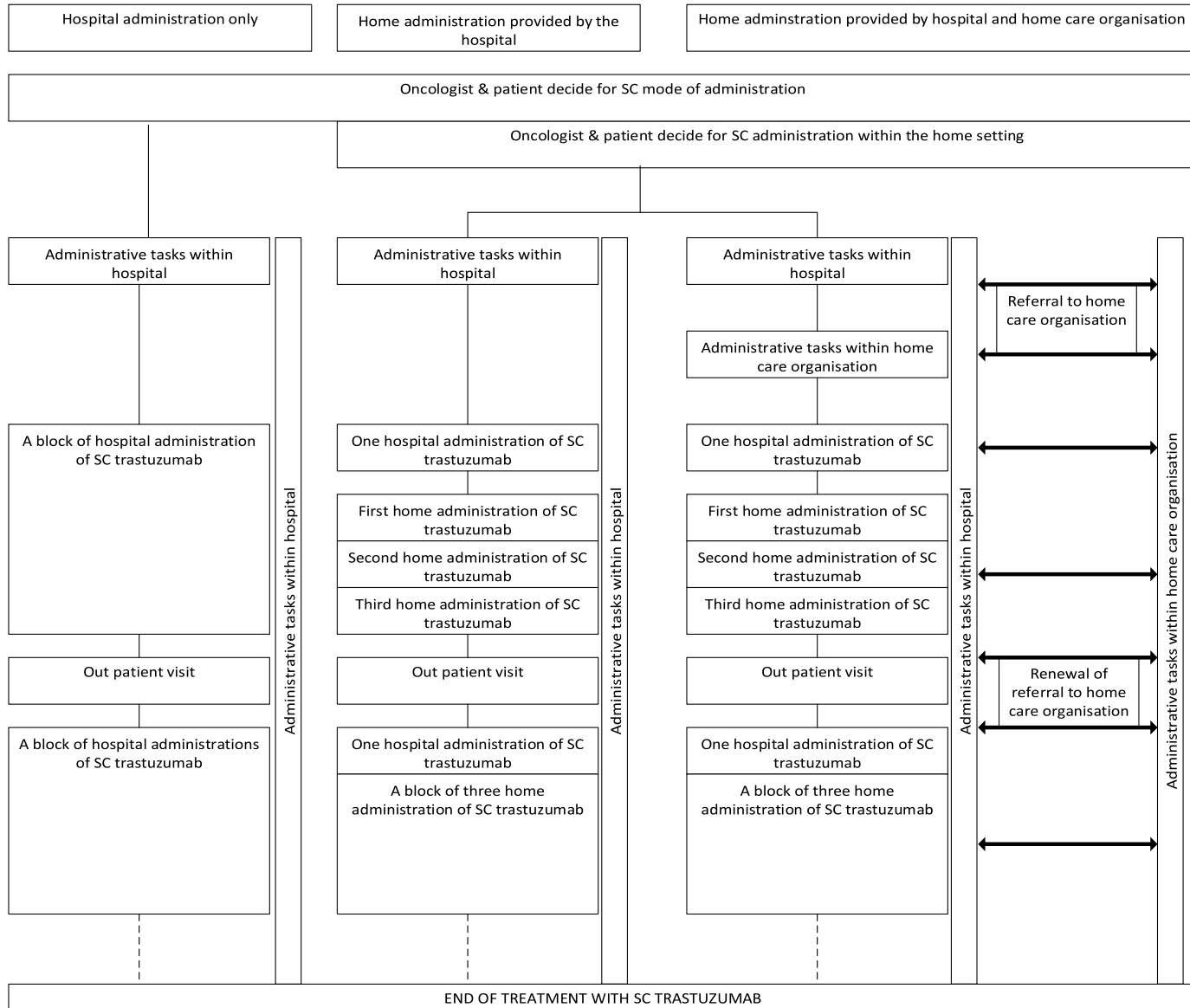

Supplement: Care pathways in the hospital-based and home-based setting [file mmc1.pdf]
